# Inverted CD8 T-Cell Exhaustion and Co-Stimulation Marker Balance Differentiate Aviremic HIV-2-Infected From Seronegative Individuals

**DOI:** 10.3389/fimmu.2021.744530

**Published:** 2021-10-12

**Authors:** Lydia Scharf, Christina B. Pedersen, Emil Johansson, Jacob Lindman, Lars R. Olsen, Marcus Buggert, Sten Wilhelmson, Fredrik Månsson, Joakim Esbjörnsson, Antonio Biague, Patrik Medstrand, Hans Norrgren, Annika C. Karlsson, Marianne Jansson, the SWEGUB CORE Group

**Affiliations:** ^1^ Department of Laboratory Medicine, Karolinska Institutet, Stockholm, Sweden; ^2^ Section for Bioinformatics, Department of Health Technology, Technical University of Denmark, Kongens Lyngby, Denmark; ^3^ Center for Genomic Medicine, Copenhagen University Hospital, Copenhagen, Denmark; ^4^ Department of Laboratory Medicine, Lund University, Lund, Sweden; ^5^ Department of Clinical Sciences Lund, Lund University, Lund, Sweden; ^6^ Center for Infectious Medicine, Department of Medicine Huddinge, Karolinska Institutet, Stockholm, Sweden; ^7^ Department of Translational Medicine, Lund University, Lund, Sweden; ^8^ National Laboratory for Public Health, Bissau, Guinea-Bissau

**Keywords:** HIV-2, aviremic, CD8 T cell phenotypes, immune activation, T cell exhaustion, costimulation, TIGIT, CD226

## Abstract

HIV-2 is less pathogenic compared to HIV-1. Still, disease progression may develop in aviremic HIV-2 infection, but the driving forces and mechanisms behind such development are unclear. Here, we aimed to reveal the immunophenotypic pattern associated with CD8 T-cell pathology in HIV-2 infection, in relation to viremia and markers of disease progression. The relationships between pathological differences of the CD8 T-cell memory population and viremia were analyzed in blood samples obtained from an occupational cohort in Guinea-Bissau, including HIV-2 viremic and aviremic individuals. For comparison, samples from HIV-1- or dually HIV-1/2-infected and seronegative individuals were obtained from the same cohort. CD8 T-cell exhaustion was evaluated by the combined expression patterns of activation, stimulatory and inhibitory immune checkpoint markers analyzed using multicolor flow cytometry and advanced bioinformatics. Unsupervised multidimensional clustering analysis identified a cluster of late differentiated CD8 T-cells expressing activation (CD38+, HLA-DR^int/high^), co-stimulatory (CD226+/-), and immune inhibitory (2B4+, PD-1^high^, TIGIT^high^) markers that distinguished aviremic from viremic HIV-2, and treated from untreated HIV-1-infected individuals. This CD8 T-cell population displayed close correlations to CD4%, viremia, and plasma levels of IP-10, sCD14 and beta-2 microglobulin in HIV-2 infection. Detailed analysis revealed that aviremic HIV-2-infected individuals had higher frequencies of exhausted TIGIT+ CD8 T-cell populations lacking CD226, while reduced percentage of stimulation-receptive TIGIT-CD226+ CD8 T-cells, compared to seronegative individuals. Our results suggest that HIV-2 infection, independent of viremia, skews CD8 T-cells towards exhaustion and reduced co-stimulation readiness. Further knowledge on CD8 T-cell phenotypes might provide help in therapy monitoring and identification of immunotherapy targets.

## Introduction

Disease progression and AIDS development occurs at different inter-individual rates after infection with human immunodeficiency virus type 1 (HIV-1) or type 2 (HIV-2) (1). However, time to AIDS is almost twice as long during HIV-2, compared to HIV-1, infection (2, 3). The lower plasma viral load (VL) in the HIV-2-infected individuals (4–8), is believed to contribute to the slower disease progression. Furthermore, potent and sustainable humoral (9–11) and cellular (12–15) immune responses are elicited during HIV-2 infection. HIV-2 has also been shown to delay subsequent HIV-1 disease progression during dual HIV-1/2 (HIV-D) infection (16, 17), and HIV-1 cross-reactive immunity in HIV-2-infected individuals has been reported (18–21). Still, HIV-2-infected individuals, can develop immunodeficiency despite low or undetectable viremia (22), and AIDS onset occurs at higher CD4 T-cell levels (3, 23). Thus, even though HIV-2 is less aggressive compared to HIV-1, individuals infected with HIV-2 display patterns of immune pathology of different cell populations, for example myeloid, natural killer (NK), invariant natural killer T (iNKT) cells, and T-cells (22, 24–27).

Chronic systemic immune activation is a central part of the immune pathology of HIV infection and drives immune exhaustion, immune aging, as well as numerous non-AIDS comorbidities [reviewed in (28)]. In this process the memory T-cell repertoire becomes highly dysfunctional, referred to as T-cell exhaustion. In concert with the loss of function, exhausted T-cells upregulate activating receptors (HLA-DR and CD38), co-stimulatory molecule (CD226, also known as DNAM-1) and inhibitory receptors, including 2B4 (CD244), programmed cell death 1 (PD-1), and T-cell immunoglobulin and ITIM domain (TIGIT) [reviewed in (28)]. In chronic HIV-1 infection, CD8 T-cell exhaustion is linked to an intermediate transcriptional phenotype expressing high levels of Eomesodermin (Eomes) that is directly linked to co-expression of PD-1 and TIGIT (29, 30). The combined expression of these markers in progressive HIV-1 infection are implicated not only in the blunting of virus specific immune responses (30), but also in the persistence of HIV-1-infected cells (31, 32).

Increasing TIGIT-mediated inhibition of CD8 T-cells and parallel downregulation of CD226 has been reported in HIV-1 infection despite early initiation of antiretroviral therapy (ART) (15, 30). TIGIT and CD226 compete in binding to a common receptor, the poliovirus receptor (PVR/CD155). CD226 is a co-stimulatory molecule that is involved in the proliferation and differentiation of T-cells (33–35) and important for effector functions of CD8 T-cells and NK cells in HIV-1 infection (30, 36, 37). Expression patterns of TIGIT and CD226 on T-cells in HIV-2 infection are, however, unexplored.

In HIV-2 infection, disease progression in the form of CD4 T-cell depletion is associated with immune activation (HLA-DR, CD38, CD69, Fas molecules), rather than viral load (24). However, activation of CD4 and CD8 T-cells varies greatly between different individuals with HIV-2 infection, where aviremic individuals have CD4 T-cells with lower immune activation compared to those with viremia (22, 24, 38, 39). The exhaustion of activated CD8 T-cells could contribute to HIV-2 progression, including risk of AIDS and non-AIDS illnesses. Especially since maintenance of functional CD8 T-cells, i.e., evasion of exhaustion, has been linked to HIV-2 control (12, 14, 15, 40). Whether CD8 T-cell subpopulations co-expressing activation, stimulatory and inhibitory immune checkpoint markers in different patterns separate aviremic HIV-2-infected from seronegative individuals remains, however, essentially unknown.

In the current study, we aimed to identify markers distinguishing CD8 T-cell pathogenesis in viremic or aviremic HIV-2-infected individuals as compared to seronegative individuals, as well as in HIV-1 and HIV-D-infected individuals, all belonging to the same unique occupational cohort.

## Methods

### Study Participants

To define CD8 T-cell markers associated with immunopathogenesis in HIV-2-infected individuals, blood samples were obtained from study participants belonging to a well-defined, occupational cohort of police officers in Guinea-Bissau (**Table 1**) (41, 42). In addition to HIV-2 infected individuals, individuals diagnosed with HIV-1 or HIV-1/HIV-2 dual (HIV-D) infections, all either treatment-naïve or not successfully treated [with VL > 1000 RNA copies/ml (43)], were included. For comparison, successfully treated HIV-1-infected participants and HIV-seronegative individuals within the same cohort were included. Informed consent was obtained from the participants and the National Ethical Committee, Ministry of Public Health in Guinea-Bissau, and the Ethical Committee at Lund University, approved the study.

**Table 1:** Characteristics of study participants ***^a^***.

	HIV-1*^b^*	HIV-1*^c^* treated	HIV-2*^b^*	*HIV-2^d^ viremic*	*HIV-2^e^ aviremic*	HIV-D*^b,f^*	HIV seronegative
Numbers (female/male)	12 (5/7)	8 (4/4)	23 (5/18)	*9 (0/9)*	*14 (5/9)*	5 (1/4)	27 (10/17)
Age in years^g^	50 (43-54)	44 (37-56)	57 (50-60)	*57 (51-61)*	*56 (46-60)*	55 (41-59)	55 (48-61)
% CD4 T-cells of lymphocytes^g^	9.8^h^ (5.3-12.6)	30.2 (11.9-36.8)	22.4 (14.2-34.7)	*14.2 (9.8-15.7)*	*33.4 (25.1-43.2)*	20.4 (8.0-30.2)	39.3 (35.5-44.9)
CD4 T-cells (cells/µl)^g^	213^h^ (125-298)	566 (260-864)	497 (302-806)	*232 (144-459)*	*636 (416-998)*	418 (195-1009)	918 (859-1204)
Viral load (copies/mm^3^)^g^,^i^	35964 (9740-56652)	<75 (<75-286)	<75 (<75-1748)	*4184 (870-21849)*	*<75*	13619^j^ (2727-41034)	NA

^a^Characteristics of participants (n=77) grouped according to HIV status and treatment, where the characteristics of the HIV-2-infected individuals is given both for the total group and for the two subgroups divided according to level of viremia. ^b^HIV-1, HIV-2 or HIV-1/2 dually (HIV-D) infected individuals either naïve to treatment or receiving suboptimal ART (plasma VL>1000 RNA copies/ml); ^c^Individuals receiving ART with viral control (plasma VL < 1000 RNA copies/ml); ^d^HIV-2-infected viremic individuals either naïve to treatment (VL>75 RNA copies/ml) or receiving suboptimal ART (VL>1000 RNA copies/ml); ^e^HIV-2-infected aviremic individuals naïve to treatment (plasma VL<75 RNA copies/ml); ^f^Individuals either naïve to treatment or receiving suboptimal ART (plasma HIV-1 VL>1000 RNA copies/ml); ^g^Median (interquartile range (IQR)); ^h^Data missing for one individual; ^i^Viral load quantification limit for mono and dual infections was 75 and 135 RNA copies/ml plasma respectively; ^j^Indicated viral load (VL) for the HIV-D-infected individuals comprise the sum of HIV-1 and HIV-2 loads, where the median HIV-1 VL was 13619 (IQR 3078-41034 copies RNA/ml) and the median HIV-2 VL was <75 (IQR <75-118 copies RNA/ml); NA, not applicable.

### Sample Collection and CD4 T-Cell Level Determination

All samples were collected during a 14-day time period on site in Guinea-Bissau and shipped to Sweden. Plasma was collected using EDTA vacutainer tubes (BD Biosciences) and whole blood using Cyto-Chex BCT tubes (Streck), in which cells are preserved while inadequate for functional analysis (44). HIV status was determined as previously described (45), and confirmed using the Geenius HIV-1/2 confirmatory assay (Bio-Rad). Absolute numbers of CD4 T-cells/µl and percentage of CD4 T-cells of lymphocytes (CD4%) were determined using the FACSPresto instrument (BD Biosciences). We used CD4% as an additional marker of immunodeficiency due to findings that CD4% is a more stable disease marker than absolute CD4 T-cell count in settings with elevated pathogenic burden and comorbidities (17, 23, 46), the close correlation to markers of T-cell exhaustion in HIV-1 infection (47), and on the previous use of this marker in the studied cohort (48).

### Plasma HIV-1 and HIV-2 Viral Load

With minor modifications, HIV-1 and HIV-2 VL were determined by in-house quantitative PCR (qPCR) protocols as described (49). Briefly, viral RNA was extracted using miRNeasy micro Kit (Qiagen) and TaqMan qRT-PCR was performed using the Superscript III Platinum One Step qRT-PCR kit (ThermoFisher Scientific). The VL quantification limit was 75 RNA copies/ml plasma for HIV-1 or HIV-2 mono-infected, and 135 RNA copies/ml plasma for HIV-D-infected.

### Flow Cytometry

Directly upon arrival from Guinea-Bissau all whole blood samples, collected in Cyto-Chex BCT tubes (Streck), were processed and analyzed on a BD Fortessa instrument (BD Biosciences) as previously described (22). Likewise, it was confirmed that the expression patterns of markers were equivalent between Cyto-Chex-stabilized blood and fresh blood samples (data not shown). In brief, the blood samples were incubated in Lysis buffer (BD Biosciences) at a ratio of 1:6 for 5 min before centrifugation and washing in PBS/FCS (2%). After treatment with DNase (6 U/ml), the cells were extracellularly stained in PBS/EDTA (2mM) for 10 min at 37°C and further 20 min at room temperature. Permeabilization was performed with the FoxP3 kit (eBiosciences) prior to 60 min intracellular staining. Antibodies used for staining of extracellular (2B4, CCR7, CD3, CD4, CD8, CD14, CD19, CD38, CD45RO, CD226, CXCR5, HLA-DR, PD-1 and TIGIT) and intracellular (Eomes) antigens, clones, fluorophores and suppliers are listed in **Supplementary Table S1**. The cells were resuspended in Cytofix Buffer (BD Biosciences) and analyzed on a BD Fortessa.

### Quantification of Plasma Inflammatory Markers

Plasma concentrations of interferon gamma-induced protein 10 (IP-10, also known as CXCL10), soluble CD14 (sCD14), and β2-microglobulin (B2M), which are plasma markers previously linked to chronic immune activation and inflammation, viremia and disease progression in HIV-1 and HIV-2 infections (50–52), were analyzed in plasma of HIV-2-infected individuals using the Magnetic Luminex assay (R&D Systems Inc.) on the Bio-Plex 200 platform (Bio-Rad Laboratories Inc.) according to manufacturer’s instructions. Plasma was diluted 1:2, 1:600 and 1:4000 for quantification of IP-10, sCD14 and B2M, respectively.

### Data Processing and Statistical Analysis

The compensated flow cytometry dataset was minimized by isolating CD8 T-cells (CD3+CD8+) out of single CD14-CD19- lymphocytes using the FlowJo 2 LLC software (version 10.5.3). For FlowSOM analysis, the dataset was limited to HIV-infected individuals. One HIV-1 and two HIV-2 aviremic individuals were subsequently excluded due to technical issues and quality of flow cytometry data. CD8 T-cell populations from the remaining individuals were then clustered using the FlowSOM algorithm (53) in R (54). Data for all cells (*n *= 3,232,181) across all samples (*n *= 45) were loaded, logicle transformed and scaled using the FlowSOM() function. A 10x10 grid was used for clustering based on the expression of ten markers: CD45RO, CCR7, HLA-DR, CD38, 2B4, PD-1, TIGIT, Eomes, CD226 and CXCR5. We set rlen = 100 and compensate = FALSE, as compensation had already been applied. We finally used maxMeta = 45, such that the final number of meta-clusters was determined by the FlowSOM() function itself.

This was followed by statistical analyses of differences between groups using a negative binomial generalized linear model from the edgeR package (55). Group-wise comparisons were individually FDR-controlled using the Benjamini-Hochberg method. Heatmaps showing cluster distribution per sample and median expression of the clustering markers were generated using ComplexHeatmap (56). The UMAP was based on scaled expression values of the ten clustering markers and generated using the uwot package (57). It is based on a sampling of 50,000 cells per HIV status category, with approximately equal sampling across samples within each of the categories. The ggplot2 package (58) was used for both the UMAP and density plots, which depict the expression distributions for each clustering marker across all cells in a given cluster.

For subsequent analysis using GraphPad PRISM, we used a manually gated dataset including data from all study participants. Here, memory CD8 T-cells were identified from CD3+CD8+, single CD14-CD19- lymphocytes, followed by exclusion of naïve CCR7+CD45RO- T cells. The frequencies of the analyzed cell populations obtained by Boolean gating were guided by “fluorescence minus one” (FMO) controls in FlowJo. Differences between study participants were analyzed using Mann-Whitney U test, comparing two groups, and Kruskal Wallis test with Dunn´s post-test, comparing more than two groups. Spearman rank correlation analysis was used to test correlations between CD8 T-cell populations and CD4 levels, VL and plasma inflammation markers. Manually gated co-expression patterns were analyzed using permutation tests provided in SPICE (version 6.0) (59).

## Results

### Study Participants

To characterize CD8 T-cell phenotypes in HIV-2-infected individuals, participants belonging to an occupational cohort in Guinea-Bissau (41, 42) were enrolled (**Table 1**). The HIV-2-infected participants (n=23), all either treatment naive or unsuccessfully treated, were further subdivided into viremic (n= 9) and aviremic (n=14) individuals, based on the plasma VL quantification level of 75 RNA copies/ml. In addition, HIV-1 (n=12) or HIV-D-infected (n=5), either treatment naive or unsuccessfully treated, as well as successfully treated HIV-1-infected (n=8) with VL < 1000 RNA copies/ml, and HIV seronegative (n=27) individuals belonging to the same cohort were studied. Both median CD4% and absolute CD4 T-cell counts were found to be higher in the seronegative individuals as compared to the viremic HIV-2 individuals (p<0.001), and in the aviremic HIV-2 compared to the viremic HIV-2 group (p=0.007 and p=0.024, respectively), whereas no statistical difference were observed between the HIV-2 aviremic and the seronegative participants (**Figures S1A, B**). A statistical difference in CD4 T-cell levels, with lower median CD4% and absolute CD4 T-cell count, was however observed between the treated HIV-1 group as compared to the seronegative participants (p=0.019 and p=0.031, respectively; **Figures S1C, D**). Additional characteristics of the study participants are depicted in **Table 1**.

### Differences in the Phenotype of CD8 T-Cell Populations Between Aviremic and Viremic HIV-2 Infection Identified by Unsupervised Clustering

It has previously been demonstrated that HIV-1 specific CD8 T-cells display an exhausted phenotype that is only partially restored by ART (29, 30). We have recently linked this phenotype to the TIGIT/CD226 axis in HIV-infection (30). To which extent the TIGIT/CD226 axis is affected by HIV-2 infection, particularly in relation to viremia has not been investigated.

**Figure 1:**
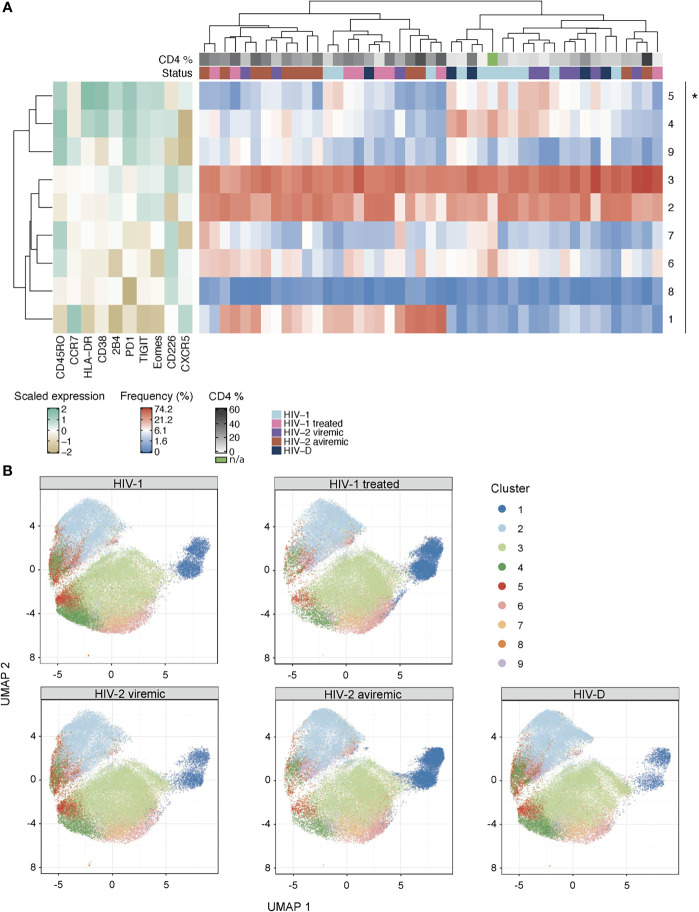
CD8 T-cell clusters identified by FlowSOM algorithm. **(A)** Heatmap illustrating CD8 T-cell clusters identified by unsupervised clustering using the FlowSOM algorithm. The cells were clustered according to their expression of CD45RO, CCR7, HLA-DR, CD38, 2B4, PD-1, TIGIT, Eomes, CD226 and CXCR5. The median expression level for each marker within the clusters is shown in turquoise-brown gradient in the left panel. The red-blue heatmap indicates the frequency of each cluster per subject, with patients being hierarchically clustered based on cluster distributions. CD4% and HIV status, i.e. HIV-1, HIV-1 treated, HIV-2 viremic, HIV-2 aviremic and HIV-1/2 (HIV-D) are shown on top of the heatmap for each patient. One HIV-1 and two HIV-2 aviremic individuals were excluded from the FlowSOM analysis due to technical issues and quality of flow cytometry data. To the right, cluster identity and asterisks (*) indicating the cluster with statistically significant differences identified between aviremic and viremic HIV-2 infection. Statistical differences were determined using a negative binomial generalized linear model and group-wise comparisons were individually FDR-controlled using the Benjamini-Hochberg method. **(B)** UMAP generated using a sample of 50,000 cells from each HIV status category (a total of 250,000 cells) based on scaled expression of the ten clustering markers. Samples within each category were sampled approximately equally. The plot is faceted by HIV status and colored by FlowSOM cluster.

First, unsupervised clustering analysis using FlowSOM was used to enable visualization of CD8 T-cell population expressing any combination of the assessed markers that delineated the HIV groups. The analysis revealed nine distinct CD8 T-cell clusters (**Figures 1A, B**). One of these clusters, cluster 5, showed statistical differences between aviremic and viremic HIV-2-infected study participants (**Figure 1A**). Cluster 5 consisted of late differentiated (CD45RO+, CCR7int/-) CD8 T-cells expressing a combination of activation (CD38+, HLA-DRint/high), inhibitory receptors (2B4+PD-1+TIGIT+), as well as Eomes (**Figure 1A** and **Figure S2**). Moreover, this cluster comprised a mixed population of CD226+ and CD226- cells (**Figure S2**). In HIV-1 infection, the combined expression of CD38, HLA-DR, and PD-1 has been shown to delineate T-cell pathogenesis distinguishing infected from healthy individuals (47). Similarly, although expression of Eomes is associated with memory formation, the severity of CD8 T-cell exhaustion has been linked to the expression of Eomes (60). In chronic viral infections, high expression of Eomes is a hallmark of T-cell exhaustion (29, 60, 61).

**Figure 2:**
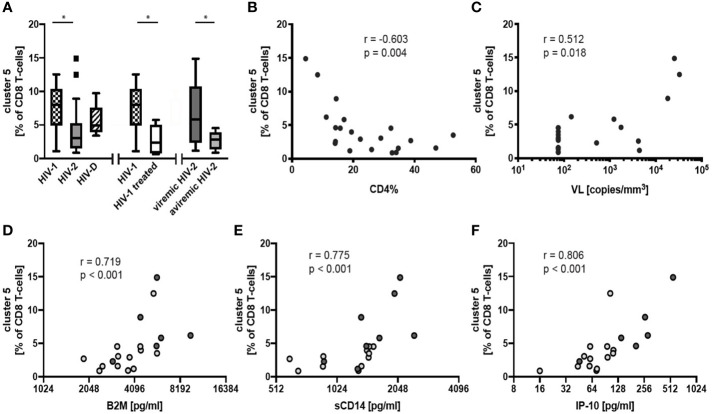
Frequencies of FlowSOM identified CD8 T-cell cluster 5 in HIV-1, HIV-2 and dually HIV-1/2 (HIV-D) infected individuals. **(A)** Tukey box plots of frequencies of CD8 T-cell cluster 5 comparing i) treatment naïve or unsuccessfully treated HIV-1 (n = 11), HIV-2 (n = 21) and HIV-D (n = 5), ii) successfully treated HIV-1 (n = 8) and treatment naïve or unsuccessfully treated HIV-1 (n = 11), iii) viremic (n = 9) and treatment-naïve aviremic (n = 14) HIV-2-infected individuals. Differences between two and three groups were determined with Mann-Whitney and Kruskal-Wallis with Dunn´s post-test, respectively. Correlations between frequency of cluster 5 in HIV-2-infected individuals and **(B)** CD4%, **(C)** plasma viral load (VL), and concentrations of inflammatory markers, **(D)** beta-2 microglobulin (B2M), **(E)** soluble CD14 (sCD14) and **(F)** IP-10, in plasma (aviremic lighter circles and viremic darker circles), according to Spearman´s rank correlation. *p < 0.05.

In HIV-2 infection, proportions of cluster 5 not only differentiated the viremic and aviremic individuals (p= 0.034; **Figure 2A**) but correlated also with disease progression in the form of declining CD4% (r = -0.603, p = 0.004; **Figure 2B**) and CD4 count (r = -0.580, p = 0.006; **Figure S3**), as well as elevated VL (r = 0.512, p = 0.018; **Figure 2C**). Moreover, increased frequencies of cluster 5 correlated with elevated plasma levels of inflammation markers, including B2M (r = 0.719, p<0.001), sCD14 (r = 0.775, p<0.001) and IP-10 (r = 0.806, p<0.001) (**Figures 2D–F**). Thus, our results indicate that cluster 5 represents CD8 T-cells with an exhausted phenotype that is clearly linked to immunopathogenesis in HIV-2 infection.

Furthermore, in treatment naïve or not successfully treated individuals, cluster 5 was more frequent in the participants with HIV-1, compared to HIV-2, infection (p = 0.027), while the frequency of these cells in HIV-D-infected participants was not different, when compared to the other groups (**Figure 2A**). Moreover, along level of viremia, lower frequencies of cluster 5 were observed in successfully treated HIV-1 infection as compared to HIV-1-infected not receiving such treatment (p = 0.003) (**Figure 2A**).

Of note, both cluster 1 and cluster 4 differentiated the aviremic and viremic HIV-2-infected groups in direct comparisons (p = 0.049 and p= 0.049 respectively) but did not differ significantly in the multiple FlowSOM comparison including all HIV-infected groups (data not shown). Cluster 1 represents a population negative for the investigated T-cell activation and immune checkpoint markers, while cluster 4, like cluster 5, consists of a population of cells expressing activation and checkpoint markers (**Figure 1A** and **Figure S2**).

In summary, unsupervised multidimensional clustering suggests that late differentiated and exhausted CD8 T-cells differentiate viremic and aviremic HIV-2 infections and are elevated with CD4 T-cell depletion and systemic inflammation.

### A Highly Activated and Exhausted CD8 T-Cell Population Distinguishes Both Aviremic and Viremic HIV-2-Infected Individuals From Seronegative Individuals

**Figure 3:**
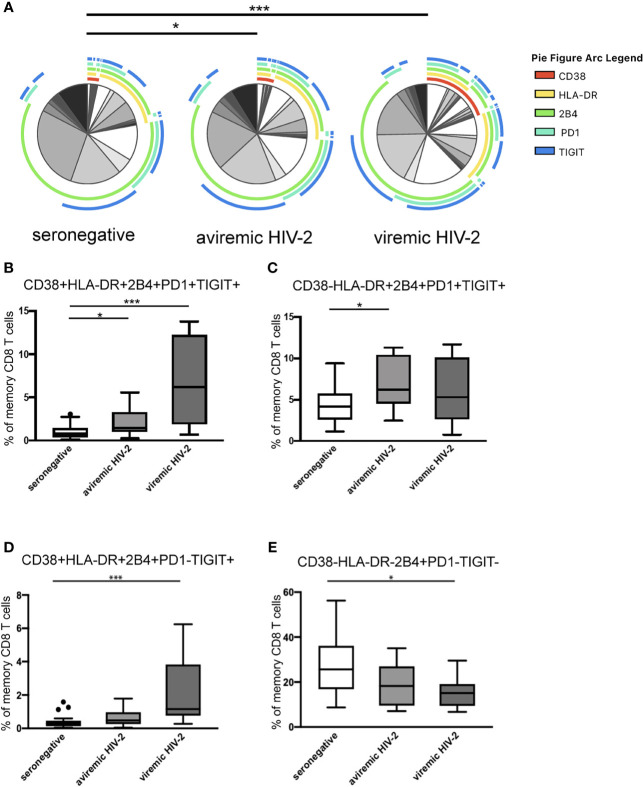
Frequencies of memory CD8 T-cells co-expressing activation and inhibitory checkpoint markers. **(A)** Pie-charts illustrating SPICE analysis, where arcs show the analyzed markers and the pie-sectors the relative frequency of the memory CD8 T-cell populations positive for the markers, and tukey box plots illustrating frequencies of specific memory CD8 T-cell populations being **(B)** CD38+HLA-DR+ 2B4+PD-1+TIGIT+, **(C)** CD38-HLA-DR+ 2B4+PD-1+TIGIT+, **(D)** CD38+HLA-DR+ 2B4+PD-1-TIGIT+ or **(E)** CD38-HLA-DR-2B4+PD-1-TIGIT- in seronegative participants (n = 27), treatment-naïve aviremic (n = 14) and viremic (n = 9) HIV-2-infected individuals. Permutation test was performed in the SPICE analysis and Kruskal Wallis test with Dunn´s post-test was performed for statistical comparisons of specific CD8 T-cell populations between the groups. *p < 0.05 and ***p < 0.001.

To evaluate the impact of aviremic HIV-2 infection on T-cell pathogenesis, we next compared the HIV-2 groups in relation to the seronegative individuals. Guided by the results obtained by the unsupervised clustering analysis and previous knowledge in the HIV-field linking the combined expression of activation and inhibitory receptors to T-cell exhaustion, senescence and health (22, 30, 47), we investigated CD8 T-cell populations expressing different combinations of CD38, HLA-DR, 2B4, PD-1, and TIGIT. According to the permutation analysis statistically significant differences were found between the seronegative control group and both the aviremic and viremic HIV-2-infected group (p = 0.050 and p < 0.001) (**Figure 3A**). More in depth analyses revealed that differences between the HIV-2-infected groups and seronegative individuals could be traced back to four different population. First, a cell population positive for all five analyzed markers (CD38+HLA-DR+2B4+PD-1+TIGIT+) (p=0.042; **Figure 3B**), best resembling the expression pattern of cluster 5 and separating both the aviremic and viremic HIV-2 groups from the seronegative participants. Similarly, the cell population solely lacking CD38, also separated the aviremic HIV-2 from the seronegative individuals (p=0.031; **Figure 3C**), but not the seronegative from the viremic HIV-2. Based on the co-expression of PD-1 and TIGIT that is linked to increased inhibitory function (62, 63), and impaired HIV-1 specific T-cell responses (30), these populations resemble exhausted T-cells. The third population, a cell population lacking PD-1 (CD38+HLA-DR+2B4+PD-1-TIGIT+) was pronounced in the group of viremic HIV-2 infected participants (p<0.001; **Figure 3D**) but did not distinguish the aviremic HIV-2 from the seronegative. In chronic viral infection, expression levels of PD-1 can determine whether T-cell exhaustion is reversible, as is the case for cells with low, but not high levels of PD-1 (64, 65). In addition, expression of PD-1 together with CD38 and CD101 are known to be permanently dysfunctional populations (66), suggesting that the populations described may represent different stages of T-cell exhaustion (67, 68), needing further attention. Moreover, a cell population only expressing 2B4 (CD38-HLA-DR-2B4+PD-1-TIGIT-) was less frequent in viremic HIV-2-infected as compared to the seronegative individuals (p = 0.022; **Figure 3E**). 2B4 can act both as a stimulatory and inhibitory receptor (69), and synergize with CD226 in a co-stimulatory capacity (70) and was further evaluated together with CD226, PD-1 and TIGIT.

When performing similar comparisons including the HIV-1 groups, the permutation analysis showed that frequencies of CD8 T-cells expressing activation and exhaustion markers differed comparing the seronegative group and the viremic HIV-1 group (p<0.001), while no such difference was seen between seronegative group and the treated HIV-1 group (**Figure S4A**). Detailed analyses further revealed that none of the activated and exhausted CD8 T-cell populations, found to be elevated in the treatment naïve aviremic HIV-2 group (**Figures 3B, C**), were increased in the successfully treated HIV-1-infected individuals compared to the seronegative group (**Figures S4B, C**).

Taken together, these findings suggest that frequency of activated and exhausted cells within the memory CD8 T-cell compartment are elevated in HIV-2-infected individuals, independent of viremia, while the level of these cells are normalized in persons with successfully treated HIV-1-infection.

### CD8 T-Cell Exhaustion Parallels Downregulation of CD226 in Both Viremic and Aviremic HIV-2 Infection

CD226 is a marker conferring receptiveness for co-stimulation that has been shown to compete with the immune inhibitory receptor TIGIT for binding to the shared ligand (71). Thus, since cluster 5 included a mix of cells expressing, or not expressing, CD226 (**Figure 1**), we next analyzed expression patterns of CD226 together with 2B4, PD-1 and TIGIT on memory CD8 T-cells. Significant differences between the seronegative control group and both the HIV-2 aviremic and viremic group were revealed by the permutation test (p=0.007 and p<0.001, respectively; **Figure S5A**). Furthermore, the permutation test also showed that the frequencies of CD8 T cells expressing these markers differed comparing the seronegative participants and the viremic HIV-1 group (p<0.001), and also comparing the treated and viremic HIV-1 groups (p=0.045) (**Figure S5B**).

**Figure 4:**
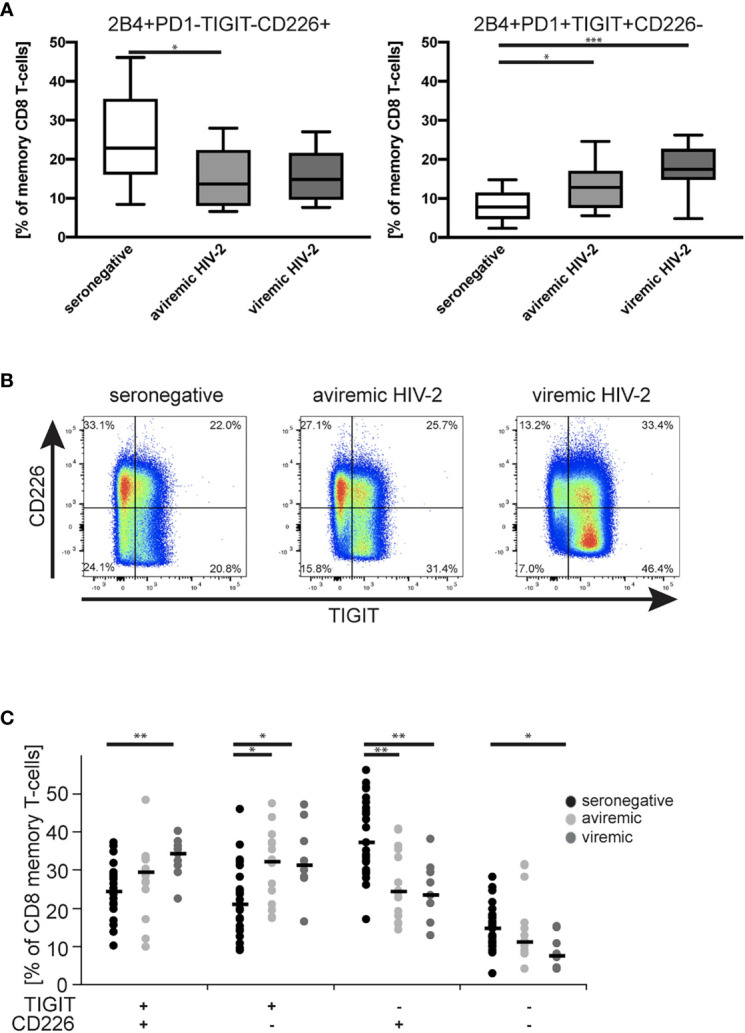
Frequencies of memory CD8 T-cells co-expressing different patterns of stimulatory and inhibitory checkpoint markers. Tukey box plots illustrating frequencies of specific memory CD8 T-cell populations being **(A)** 2B4+PD-1-TIGIT-CD226+ or 2B4+PD-1+TIGIT+CD226- in seronegative participants (n = 27) and treatment naïve aviremic (n = 14) and viremic (n = 9) HIV-2-infected individuals. **(B)** Examples of flow cytometry plots depicting the expression of CD226 and TIGIT on memory CD8 T-cells of representative study participants. **(C)** Expression patterns of TIGIT and CD226 on memory CD8 T-cells in the study participants depicted above. The black bars depict medians. Statistical differences between groups were analyzed using Kruskal-Wallis test and Dunn´s post-test. *p < 0.05, **p < 0.01 and ***p < 0.001.

More specifically, the frequency of 2B4+PD-1-TIGIT-CD226+ CD8 T-cells was lower among memory CD8 T-cells of HIV-2 aviremic (p = 0.021) individuals when compared to seronegative individuals (**Figure 4A**). In contrast, the frequency of highly exhausted (2B4+PD-1+TIGIT+CD226-) CD8 T-cells increased gradually in relation to HIV-2 viremia and was significantly higher in both the HIV-2 aviremic and viremic groups compared to the seronegative controls (p = 0.043 and p < 0.001 respectively) (**Figure 4A**).

To focus on the TIGIT/CD226/PVR network more specifically, we limited the analysis to the expression of TIGIT and CD226. As exemplified in **Figure 4B**, a visually striking skewing of TIGIT and CD226 expression patterns was noted when comparing individuals of the three study groups. The population frequencies of TIGIT+CD226+ double positive and TIGIT-CD226- double negative CD8 T-cells both differed between the viremic HIV-2-infected and seronegative individuals (p = 0.003 and p = 0.010, respectively) (**Figure 4C**). In addition, the frequency of the stimulation-receptive TIGIT-CD226+ CD8 T-cells was decreased in both aviremic (p = 0.003) and viremic (p = 0.004) HIV-2-infected individuals when compared to the seronegative group. Conversely, exhausted and stimulation-unreceptive TIGIT+CD226- CD8 T-cells were increased in both aviremic (p = 0.012) and viremic (p = 0.015) individuals compared to the seronegative controls (**Figure 4C**). Of note, we found no significant differences in the CD8 T-cell differentiation phenotypes of TIGIT+CD226- and TIGIT-CD226+ CD8 T-cells (**Figure S5C**) between the groups.

In summary, the expression pattern of TIGIT and CD226 suggests that both aviremic and viremic HIV-2 infection skews the expression of the regulatory TIGIT/CD226/PVR receptor network on CD8 T-cells towards exhaustion, away from the stimulation-receptive state seen in the HIV seronegative population.

## Discussion

Our study underscores that immunopathology, strongly driven by immune activation (CD38, HLA-DR) and immune checkpoint (2B4, PD-1, TIGIT) markers, distinguishes aviremic HIV-2-infected individuals from the seronegative population as well as the viremic HIV-2-infected group. Furthermore, the presented novel results highlight that CD8 T-cell co-stimulation receptiveness (*e.g.*, by CD226) of primarily viremic and to a lesser degree aviremic HIV-2-infected individuals is blunted compared to seronegative individuals.

Guided by unsupervised clustering analysis of the total CD8 T-cell population including all HIV-infected groups, we conclude that a cluster defined by activated (CD38 and HLA-DR) and exhausted (2B4, PD-1 and TIGIT) late differentiated memory CD8 T-cells expressing Eomes distinguish aviremic from viremic HIV-2-infected individuals. Synergistic upregulation of PD-1 and TIGIT is linked to increased inhibitory function (62, 63), poor functionality of HIV-1 specific T-cell responses (30) and is inversely correlated with CD4% and CD4/CD8 ration in HIV-1 infection (72), suggesting that this population resembles highly exhausted CD8 T-cells. In line with our hypothesis, the prevalence of this CD8 T-cell population was linked to both increased viremia and declining CD4 T-cell levels, as well as augmented levels of plasma inflammation markers, within the HIV-2-infected participants.

Targeted analysis, including comparison to seronegative control individuals, disclosed that aviremic HIV-2-infected individuals have elevated frequencies of CD8 T-cells co-expressing activation and inhibitory receptors compared to the seronegative group. This is in line with our previous observation showing increased expression of CD38, HLA-DR and PD-1 on memory CD4 T-cell populations in aviremic HIV-2 infection (22). Thus, both the CD4 and the CD8 T-cell compartments appear to display pathological phenotypes independent of the viremia detected in peripheral blood. An explanation could possibly be the long duration of the HIV-2 infection, since many of the aviremic study participants had been infected for more than 20 years (data not shown). In untreated HIV-1 infection progressively increased expression of PD-1 on both CD4 and CD8 T-cell subsets have been demonstrated in patients with long-follow-up (31). Alternatively, low-grade viremia, not detected by the methods used in the current study, could exhaust the HIV-2 specific CD8 T-cells. Due to the cell preservative used for sample collection in the current study, we were not able to distinguish HIV-specific from bulk CD8 T-cells by inclusion of functional markers. However, it was previously demonstrated that elevated frequencies of PD-1 and 2B4 expression are especially abundant on virus-specific CD8 T-cells in HIV-2 infection (15).

While the current results suggest that successful treatment of HIV-1 result in normalized levels of activated and exhausted CD8 T-cells, we have in another cohort found that memory CD8 T-cells expressing TIGIT, PD1, CD160 and 2B4 are increased in treated HIV-1 infection as compared to seronegative (30). This discrepancy might be linked to the limited sample size of the treated HIV-1-infected group in the current study. Alternatively, the background frequency of activated and exhausted CD8 T-cells in the seronegative population in the geographic setting of the studied cohort might be elevated, as a consequence of high pathogenic burden in the area. The phenotypes, as detected by the unsupervised clustering or the specific populations, of the pathogenic memory CD8 T-cells in HIV-D-infected individuals were not possible to distinguish from the HIV-1 or the HIV-2-infected groups (data not shown). However, identification of small differences in the CD8 T-cell populations between the HIV-D infected and the other groups, might also been hampered by the limited number of samples. Thus, further studies are merited to characterize in more detail the phenotype of CD8 T-cells in dual infection.

Based on the noted high expression of TIGIT, we further investigate the role of the TIGIT/CD226 network, not previously investigated in HIV-2 infection. Together with TIGIT, the CD226 receptor linked to receptiveness of memory CD8 T-cells to co-stimulation, is part of a network interacting on several levels. TIGIT outcompetes CD226 in binding to a shared ligand, PVR (62), and prevents homodimerization of CD226, consequently disrupting downstream signaling (73). Here, we found clear evidence that HIV-2 infection skews this network towards inhibition, as TIGIT+CD226- CD8 T-cells are increased in both aviremic and viremic HIV-2-infected individuals. A similar trend was observed in double positive TIGIT+CD226+ cells, where the inhibiting receptor TIGIT presumably outcompetes co-stimulation by CD226 (62, 71) when expressed at comparable levels. In contrast, the TIGIT-CD226+ expression pattern that unquestionably allows for co-stimulation, was significantly less frequent among both aviremic and viremic HIV-2-infected individuals, suggesting that despite low HIV-2 plasma viremia, skewing of the TIGIT/CD226/PVR axis may occur, in a manner similar to that seen in HIV-1-infected individuals on virus-controlling ART and HIV-1 elite controllers (30). While recent advances in cancer immunotherapy showed synergistic effects of simultaneous blockade of PD-1 and TIGIT (63), subsequent studies demonstrated the requirement of CD226 expression for this approach (74). Thus, efficient use of HIV cure strategies involving immune checkpoint blockade could be challenging in individuals where CD226 expressing CD8 T-cell populations are reduced, including both viremic and aviremic HIV-2-infected individuals.

Unlike the other immune checkpoint markers in this study, 2B4 has a dual function as stimulatory as well as inhibitory receptor, depending on the context of available ligands (70), which has to be taken in consideration when interpreting expression patterns. A synergistic effect of CD226 and 2B4 inducing cytotoxicity and cytokine secretion in NK cells (75) demonstrates a connection between these receptors on cytotoxic cells. Interestingly, our analysis of CD8 T-cells expressing immune checkpoint markers in combination with CD226 revealed that the 2B4+PD-1-TIGIT-CD226+ population was decreased in aviremic HIV-2 infection when compared to seronegative controls. In contrast, the population expressing all three immune checkpoint markers but not CD226 was elevated in both HIV-2-infected groups. Because of the dual function and dependence on the context, this may result in reduced capacity to respond to both HIV and co-infections in aviremic HIV-2 infection.

Recently, Hønge et al. presented data regarding the co-stimulator CD28, which was less frequently expressed on CD8 T-cells of aviremic HIV-2-infected individuals than seronegative control individuals (76). Together with our data on CD226, 2B4 and their co-expression with other surface markers, this suggests a universal deterioration of CD8 T-cells in aviremic HIV-2 infection when compared to the HIV-seronegative background population. Here, we show that skewing of memory CD8 T-cell populations towards reduced CD226 expression and elevated expression of inhibitory receptors are indicative of immunopathological changes in aviremic HIV-2 infection, despite seemingly controlled virus replication. However, several studies have identified proviral DNA and low level of viral replication in HIV-2-infected individuals with undetectable plasma VL (5, 77), providing possible underlying mechanisms for the immunopathology. It is also likely that virus replication is ongoing in other compartments and that the extended infection duration contributes to the emergence of pathogenic memory CD8 T-cell subpopulations.

In conclusion, these results, taken together with our previous findings demonstrating that CD4 T-cells, NKT cells and NK cells of aviremic HIV-2 individuals display pathogenic phenotypes compared to seronegative individuals (22, 27) and the fact that the majority of HIV-2-infected individuals with follow-up indeed show disease development (3), suggest that aviremic HIV-2-infected individuals could benefit from early initiation of ART. Of note, there was no significant difference in CD4 count between aviremic HIV-2-infected and seronegative individuals in our study cohort. Thus, the differences in the here identified pathogenic CD8 T-cell populations between aviremic and viremic individuals, suggest that CD8 T-cell phenotyping could be a tool for treatment monitoring. Virally suppressed HIV-1-infected individuals on ART displayed significantly lower frequencies of activated and exhausted CD8 T-cell populations than the viremic HIV-1-infected individuals, while such population did not differ between HIV-1 on ART and the aviremic HIV-2-infected individuals. Still, the impact that ART may have on CD8 T-cell activation, exhaustion and co-stimulation in aviremic HIV-2-infected cohorts merits further investigations. This, since we envision that CD8 T-cell phenotyping in the future could be a tool for monitoring treatment success in aviremic HIV-2-infected individuals.

## Data Availability Statement

The original contributions presented in the study are included in the article/**Supplementary Material**. Further inquiries can be directed to the corresponding authors.

## Ethics Statement

The studies involving human participants were reviewed and approved by the National Ethical Committee, Ministry of Public Health in Guinea-Bissau and the Ethical Committee at Lund University. The patients/participants provided their written informed consent to participate in this study.

## Author Contributions

LS designed and conducted the flow cytometric analyses, interpreted the data, and drafted the manuscript. CP designed, conducted, and summarized the bioinformatics analyses. EJ conducted and analyzed the biomarker analyses. JL and HN coordinated the study cohort. AB was medically responsible for the study participants. JL, LO, MB, SW, FM, JE, PM, and HN contributed substantially to the study design and results interpretation, and critically reviewed the manuscript. AK and MJ jointly conceptualized the study and were shared principal investigators of the study. All authors contributed to the article and approved the submitted version.

## Funding

This work was supported by grants given by The Swedish Research Council to MJ (2016-02285, 2019-01439) and AK (K2014-57X-22451-01-5, 2017-01056, 2020-02033), and The Center for Medical Innovation to AK (20190495), Karolinska Institutet (FS-2018:0007, 2-1293/2014). LO and CP were funded by the Independent Research Fund Denmark (grant number 8048-00078A to LO).

## Conflict of Interest

The authors declare that the research was conducted in the absence of any commercial or financial relationships that could be construed as a potential conflict of interest.

## Publisher’s Note

All claims expressed in this article are solely those of the authors and do not necessarily represent those of their affiliated organizations, or those of the publisher, the editors and the reviewers. Any product that may be evaluated in this article, or claim that may be made by its manufacturer, is not guaranteed or endorsed by the publisher.
