# Supplementary material for: Inverted CD8 T-Cell Exhaustion and Co-Stimulation Marker Balance Differentiate Aviremic HIV-2-Infected From Seronegative Individuals
Source: Front Immunol. 2021 Oct 12;12:744530. doi: 10.3389/fimmu.2021.744530 (PMC8545800; doi:10.3389/fimmu.2021.744530)
Supplement: Supplementary file 1 [file DataSheet_1.pdf]

Supplementary Material

Table S1. Antibodies used in flow cytometry.

Antigen	Fluorophore	Clone	Supplier
2B4	FITC	C1.7	Biolegend
CXCR5	AF647	RF8B2	BD Biosciences
CCR7	AF700	G043H7	Biolegend
CD3	APC-H7	SK7	BD Biosciences
PD-1	BV421	EH12.2H7	Biolegend
CD14	V500	M5E2	BD Biosciences
CD19	V500	HIB19	BD Biosciences
CD8	BV570	RPA-T8	Biolegend
CD45RO	BV650	UCHL1	BD Biosciences
CD38	BV711	HIT2	Biolegend
CD226	BV786	DX11	BD Biosciences
Eomes	PE-eF610	WE1928	eBioscience
CD4	PE-Cy5.5	S3.5	Invitrogen
TIGIT	PE-Cy7	MBSA43	eBioscience
HLA-DR	BUV395	G46-6	BD Biosciences

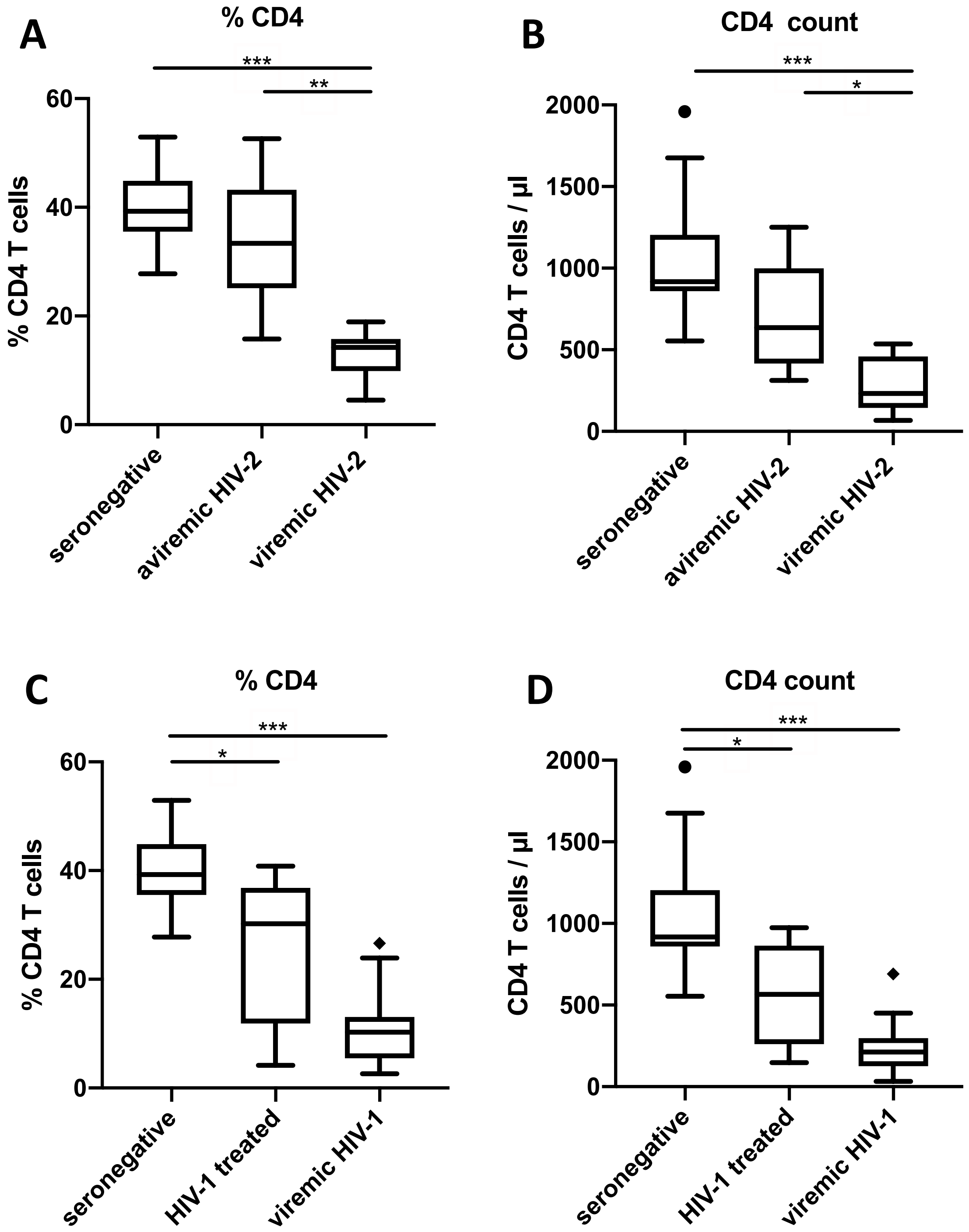

Figure S1. CD4 T-cell levels in different HIV-1 and HIV-2 infected individuals, compared to the HIV seronegative group. A) and C) percentage CD4+ T-cells among lymphocytes and B) and D) CD4+ T-cells/µl in A) and B) seronegative, aviremic and viremic HIV-2-infected individuals, and C) and D) seronegative, viremic and treated HIV-1-infected indviduals. Statistical differences calculated according to Krusal-Wallis test with Dunn´s post test. *$p>0.05$, **$p<0.01$, and ***$p<0.001$

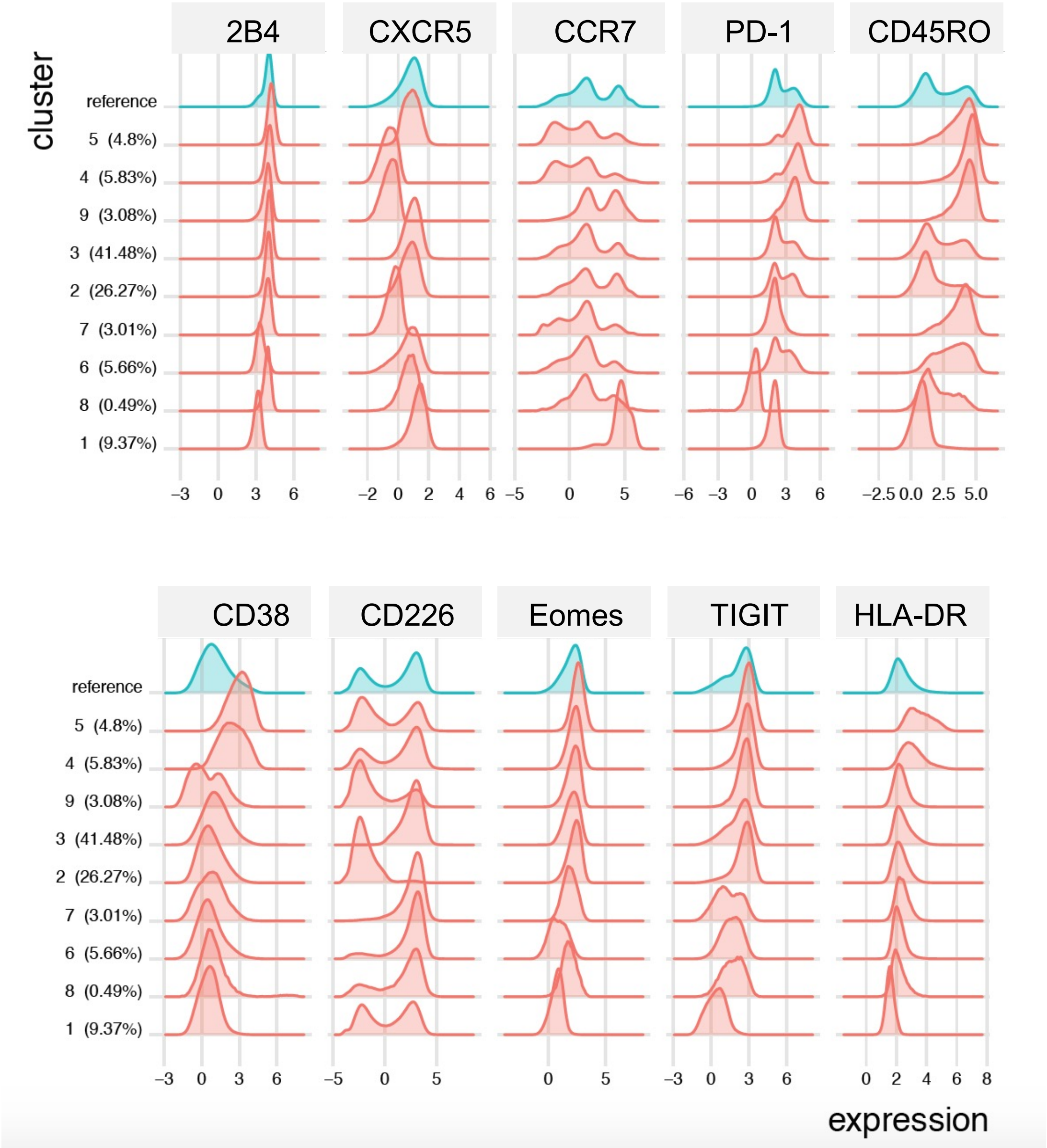

Figure S2. Expression levels of markers on CD8 T-cell clusters identified by FlowSOM algorithm. Relative expression level (mean fluorescent intensity) of markers throughout clusters identified in FlowSOM approach. The identity of each cluster and its overall frequency across samples is indicated to the left of the panel.

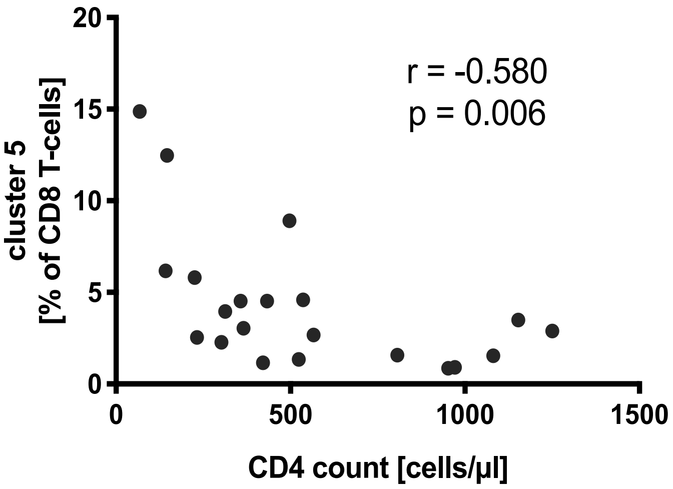

Figure S3. Frequency of cluster 5 in correlations with CD4 T-cell count. Correlation of FlowSOM identified cluster 5 with CD4 T-cell count of HIV-2-infected participants. Correlation statistics was calculated according to Mann-Whitney U-test was performed for statistical comparisons between groups, and Spearman Rank Correlations test for the correlation.

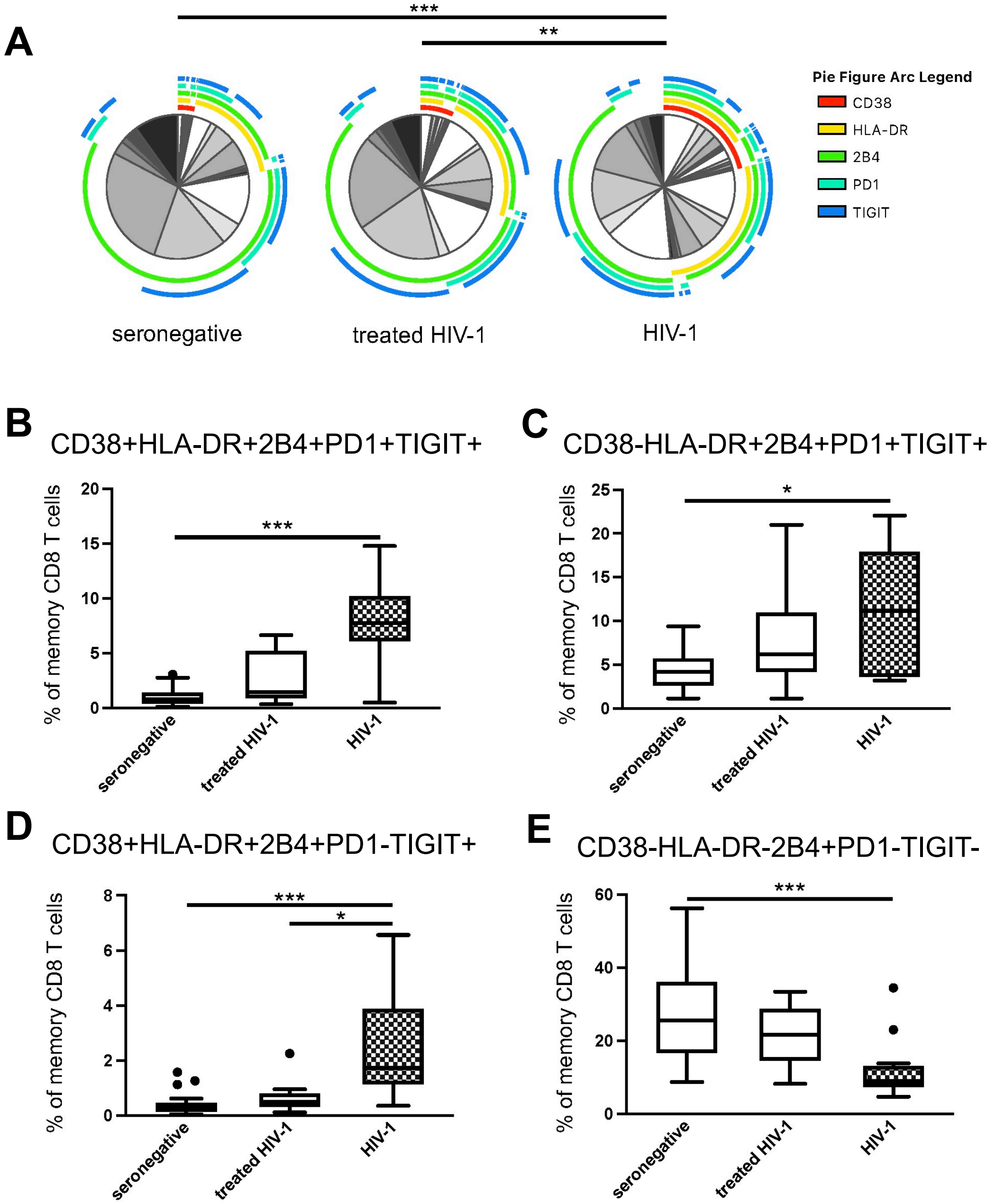

Figure S4. Frequencies of memory CD8 T-cells co-expressing activation and inhibitory checkpoint markers. (**A**) Pie-charts illustrating SPICE analysis, where arcs show the analyzed markers and the pie-sectors the relative frequency of the memory CD8 T-cell populations positive for the markers, and tukey box plots illustrating frequencies of specific memory CD8 T-cell populations being (**B**) CD38+HLA-DR+ 2B4+PD-1+TIGIT+, (**C**) CD38-HLA-DR+ 2B4+PD-1+TIGIT+, (**D**) CD38+HLA-DR+ 2B4+PD-1-TIGIT+ or (**E**) CD38-HLA-DR- 2B4+PD-1-TIGIT- in seronegative participants (n=27), successfully treated (n=8) and viremic (n=12) HIV-1-infected individuals. Permutation test was performed in the SPICE analysis and Kruskal Wallis test with Dunn´s post-test was performed for statistical comparisons of specific CD8 T-cell populations between the groups. *p<0.05 and ***p<0.001.

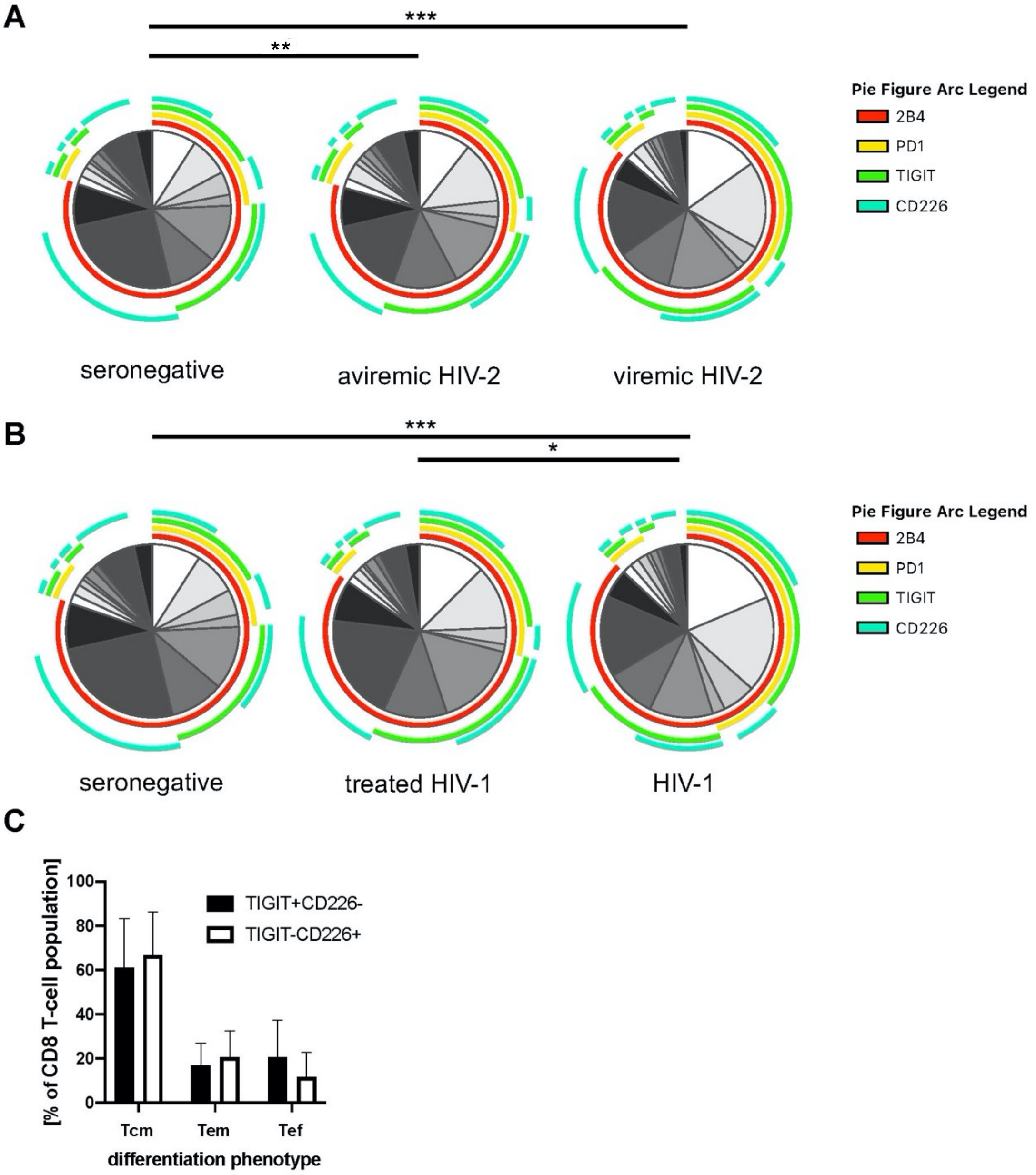

Figure S5. Combined expression of exhaustion and co-stimulation markers on CD8 T-cells. (**A**) and (**B**) Pie-charts illustrating SPICE analysis, where arcs show the analyzed markers and the pie-sectors the relative frequency of the memory CD8 T-cell populations positive for the markers of exhaustion (2B4, PD-1 and TIGIT), and co-stimulation (CD226) in seronegative participants and (**A**) viremic and aviremic HIV-2-infected individuals as well as (**B**) treated and viremic HIV-1-infected individuals. Statistical significance was determined by permutation tests provided in the SPICE software. (**C**) Proportions of differentiation phenotypes among TIGIT+CD226- and TIGIT-CD226+ memory CD8 T-cells were determined based on expression of CCR7 and CD45RO. Tcm (central memory), Tem (effector memory) and Tef (effector-like and terminal memory). Mann-Whitney U-test was performed for statistical comparisons between groups.
